# Integration of design smells and role-stereotypes classification dataset

**DOI:** 10.1016/j.dib.2021.107125

**Published:** 2021-05-08

**Authors:** Daniel Ogenrwot, Joyce Nakatumba-Nabende, Michel R.V. Chaudron

**Affiliations:** a Department of Computer Science, Gulu University, Uganda; b Department of Computer Science, Makerere University, Uganda; c Department of Mathematics and Computer Science, Eindhoven University of Technology, The Netherlands

**Keywords:** Software design, Role-stereotype, Design smells, Software quality

## Abstract

Design smells are recurring patterns of poorly designed (fragments of) software systems that may hinder maintainability. Role-stereotypes indicate generic responsibilities that classes play in system design. Although the concepts of role-stereotypes and design smells are widely divergent, both are significant contributors to the design and maintenance of software systems. To improve software design and maintainability, there is a need to understand the relationship between design smells and role stereotypes. This paper presents a fine-grained dataset of systematically integrated design smells detection and role-stereotypes classification data. The dataset was created from a collection of twelve (12) real-life open-source Java projects mined from GitHub. The dataset consists of 18 design smells columns and 2,513 Java classes (rows) classified into six (6) role-stereotypes taxonomy. We also clustered the dataset into ten (10) different clusters using an unsupervised learning algorithm. Those clusters are useful for understanding the groups of design smells that often co-occur in a particular role-stereotype category. The dataset is significant for understanding the non-innate relationship between design smells and role-stereotypes.

## Specifications Table

**Table:** 

Subject	Software Engineering
Specific subject area	This paper focuses on analysis of the association between design smells and role-stereotypes in source code.
Type of data	Raw and analysed
How data were acquired	Software projects were downloaded from GitHub using the git clone command line tool.
Data format	CSV
Parameters for data collection	The data were collected from GitHub public code repositories. All the selected projects are written in Java and licensed for redistribution within the terms of its license.
Description of data collection	Our dataset is based on five (5) desktop and seven (7) mobile (Android-based) real-life open-source Java projects mined from GitHub.
Data source location	1. https://github.com/rpax/sweethome3d 2. https://github.com/mars-sim/mars-sim 3. https://github.com/argouml-tigris-org/argouml 4. https://github.com/albfan/jEdit 5. https://github.com/bardsoftware/ganttproject 6. https://github.com/k9mail/k-9 7. https://github.com/bitcoin-wallet/bitcoin-wallet 8. https://github.com/bpellin/keepassdroid 9. https://github.com/opentripplanner/OpenTripPlanner 10. https://github.com/DrKLO/Telegram 11. https://github.com/chrislacy/TweetLanes 12. https://github.com/signalapp/Signal-Android/releases/tag/v4.69.5
Data accessibility	Raw data is publicly available at Mendeley data Data identification number: http://dx.doi.org/10.17632/6rtgxbsw68.1 Direct URL to the data: https://data.mendeley.com/datasets/6rtgxbsw68/1 The dataset citation is in Ref [4].
Related research article	D. Ogenrwot, J. Nakatumba-Nabende, and M. R. V. Chaudron. Comparison of occurrence of design smells in desktop and mobile applications. In Proceedings of the 2020 African Conference on Software Engineering (ACSE 2020), Nairobi, Kenya. CEUR Workshop Proceedings ISSN 1613-0073, vol. 2689, CEUR-WS.org, 2020. [Online] urn:nbn:de:0074-2689-6 Available: http://ceur-ws.org/Vol-2689/paper2.pdf

## Value of the Data

• We provide a fine-grained dataset derived through a systematic combination of design smells detection and role-stereotype classification data. This data is essential for researchers who are interested in studying design smells from the “lens” of role-stereotypes software system design.
• The dataset is important for software engineers to enable them to identify classes that are vulnerable to certain types of design smells based on their role-stereotypes (class responsibilities). Identifying design smells at the early stage of software design could improve software maintenance and reliability.
• The dataset is useful for software analysts who can use it to review and include new quality assurance guidelines that consider design smells and role-stereotypes.
• This dataset can provide insights to software tool builders to optimize design smell detection tools by tailoring design smell metrics to a specific project and /or role-stereotype.
• To the best of our knowledge, this is the first publicly available dataset which combines design smells detection and role-stereotypes classification data.

## 1 Data Description

**Fig. 1:**
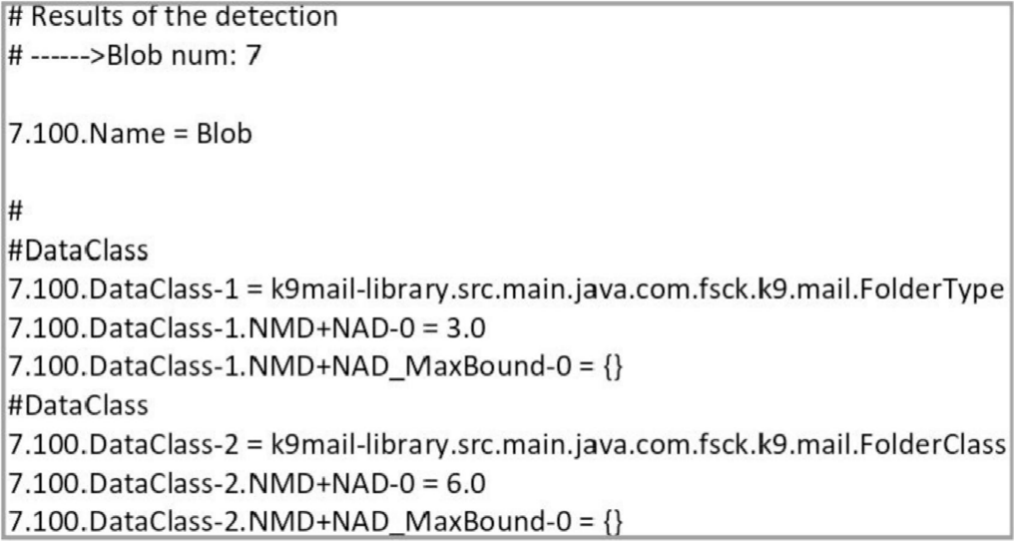
Sample content of design smells detected from source code.

**Fig. 2:**
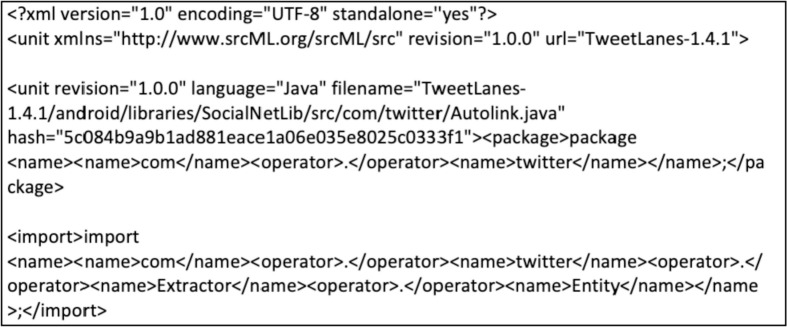
Sample output from the srcML tool preprocessing.

**Fig. 3:**
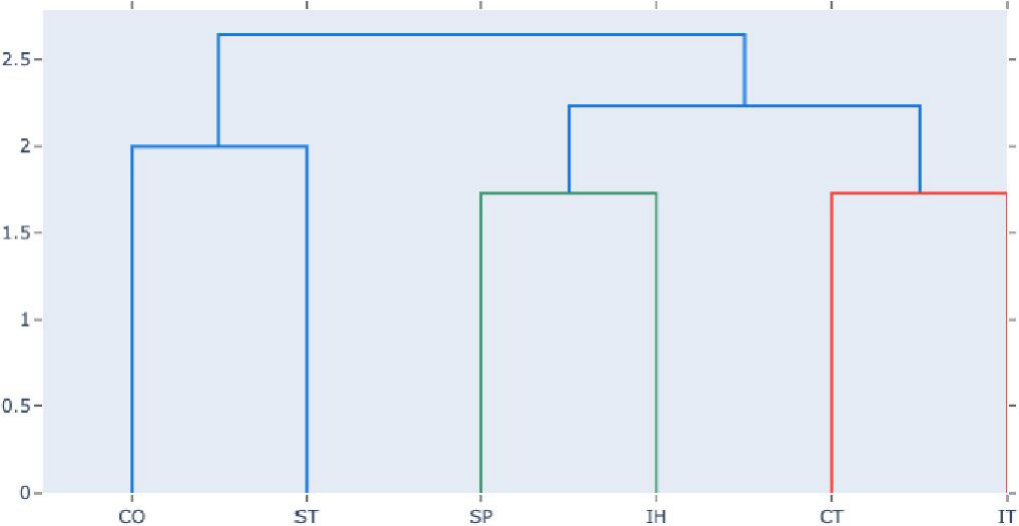
Relationship between role-stereotypes based on the common co-occuring design smells. The role-stereotypes are abbreviated as follows; Coordinator (CO), Structurer (ST), Service Provider (SP), Controller (CT) and Interfacer (IT).

We present a fine-grained dataset of systematically integrated design smells detection and role-stereotype classification data. The dataset was derived from twelve (12) real-life open-source software projects selected from GitHub public repositories. The raw dataset is publicly available as a Mendeley repository [4]. The data is described through the following tables and figures. Table 1 presents the projects used to build the dataset. Table 2 shows a fine-grained dataset which consists of the integration of design smells and role-stereotypes. Fig. 1 shows sample content of the “.ini” files in its raw format. Table 3 outlines the regular expressions used for extracting class names from the design smells files. Fig. 2, presents the sample output of role-stereotypes preprocessing tasks generated using the srcML tool. Fig. 3 shows the relationship between role-stereotypes based on the common co-occuring design smells. Table 4 presents the number of occurrences of each design smell in each category of role-stereotype. Table 5 is derived from the clustering task and it presents groups of design smells that occur in a given role-stereotype. Finally, Table 6 shows the association of design smells with role-stereotypes extracted using association rule discovery technique.

**Table 1:** Projects used to build the dataset with their version number, total lines of code (#LoC) and domain.

No.	Project	Version	#LoC	Domain
1	SweetHome3d	5.6	104,059	Desktop
2	Mars Simulation	3.1.0	255,459	Desktop
3	ArgoUML	0.35.1	177,372	Desktop
4	JEdit	5.5.0	124,164	Desktop
5	GanttProject	2.9.11	66,709	Desktop
6	K-9 Mail	5.600	93,540	Mobile
7	Bitcoin Wallet	6.31	18,079	Mobile
8	KeepassDroid	2.5.9	17,916	Mobile
9	Opentrip Planner	2.1.5	9760	Mobile
10	Telegram	6.1.1	541,694	Mobile
11	Tweet Lanes	1.4.1	25,886	Mobile
12	Text Secure	4.69.5	166,731	Mobile

**Table 2:** Sample output of the integrated design smells and role-stereotypes dataset.

No.	FullClass Path	SubClassPath	Blob	Long ParameterList	Long Method	Complex Class	...	label	Cluster
0	k9mail.src.main.java.com.fsck.k9. mailstore.BinaryAttachmentBody	k9.mailstore. BinaryAttachmentBody	1	1	0	0	...	Service Provider	5
1	k9mail.src.main.java.com.fsck.k9. activity.K9PreferenceActivity	k9.activity. K9PreferenceActivity	0	1	0	1	...	Controller	0
2	org.thoughtcrime.securesms. mms.AudioSlide	mms.AudioSlide	1	1	1	0	...	Controller	1
3	org.thoughtcrime.securesms. notifications.NotificationChannels	notifications. NotificationChannels	1	1	1	0	...	Service Provider	1
4	com.keepassdroid.database. PwDate & database.PwDate	database.PwDate	1	1	1	1	...	Interfacer	5
5	com.keepassdroid.view. FileNameView	view.FileNameView	1	1	0	1	...	Information Holder	2
6	com.keepassdroid.stream. LEDataInputStream	stream.LEDataInputStream	0	1	1	1	...	Coordinator	3
7	com.tweetlanes.android.core. view.HomeActivity	view.HomeActivity	0	1	1	1	...	Structurer	3

**Table 3:** Regular expressions used to extract the class names in each project.

No.	Project	Regular Expression
1	K-9 Mail	k9mail[a-zA-Z0-9.-]+
2	SweetHome 3D	com.eteks.sweethome3d[a-zA-Z0-9.-]+
3	Bitcoin Wallet	wallet.src.[a-zA-Z0-9.-]+
4	Argouml v0.35.1	org.argouml[a-zA-Z0-9.-]+
5	Ganttproject v2.8.11	(net.sourceforge.ganttproject.[a-zA-Z0-9.-_]+|biz.ganttproject.[a-zA-Z0-9.-_]+|org.ganttproject.[a-zA-Z0-9.-_]+|org.w3c.[a-zA-Z0-9.-_]+|com.googlecode.[a-zA-Z0-9.-_]+)
6	jEdit v5.5.0	(com.ultramixer.[a-zA-Z0-9.-_]+|org.gjt.sp.[a-zA-Z0-9.-_]+|org.jedit.[a-zA-Z0-9.-_]+)
7	Mars Simulation v3.1.0	org.mars_sim.[a-zA-Z0-9.-]+
8	Opentripplanner v2.1.5	edu.usf.cutr.opentripplanner.[a-zA-Z0-9.-_]+
9	Keepassdroid v2.5.9	(com.keepassdroid.[a-zA-Z0-9.-_]+|org.apache.commons.[a-zA-Z0-9.-_]+)
10	Textsecure v4.60.5	org.thoughtcrime.securesms.[a-zA-Z0-9.-]+
11	Telegram v6.1.1	org.telegram[a-zA-Z0-9.-_]+
12	TweetLanes v1.4.1	(org.[a-zA-Z0-9.-_]+|com.[a-zA-Z0-9.-_]+)

**Table 4:** Frequency of each type of design smell in a given role-stereotype. The highlighted cells indicate the highest value in a specific role-stereotype category.

Design Smells	Role-stereotypes					
	Service Provider	Information Holder	Interfacer	Coordinator	Controller	Structurer
SpeculativeGenerality	12	4	0	1	0	0
BaseClassKnowsDerivedClass	-	-	-	-	-	-
MessageChains	1	0	2	0	0	1
LongParameterList	402	154	149	7	29	12
SpaghettiCode	2	0	2	0	1	0
BaseClassShouldBeAbstract	24	10	5	0	1	2
LongMethod	830	92	412	0	15	20
ClassDataShouldBePrivate	103	81	57	0	13	3
TraditionBreaker	-	-	-	-	-	-
ManyFieldAttributesButNotComplex	0	5	1	0	0	0
RefusedParentBequest	102	31	7	0	0	0
SwissArmyKnife	1	2	0	0	0	1
Blob	23	45	11	5	28	14
AntiSingleton	67	27	38	0	7	4
ComplexClass	516	128	471	16	43	38
LargeClass	0	1	0	0	0	0
FunctionalDecomposition	-	-	-	-	-	-
LazyClass	211	44	11	0	1	0

**Table 5:** Groupings of design smells in each role-stereotype category generated from the POPC clusters.

No	Role-stereotypes	Design Smells Grouping	Number of Design smells
1.	Service Provider	SpeculativeGenerality, MessageChains, LongParameterList, SpaghettiCode, BaseClassShouldBeAbstract, LongMethod, ClassDataShouldBePrivate, RefusedParentBequest, SwissArmyKnife, Blob, AntiSingleton, ComplexClass, LazyClass	13
2.	Information Holder	SpeculativeGenerality, LongParameterList, BaseClassShouldBeAbstract, LongMethod ClassDataShouldBePrivate, SwissArmyKnife, Blob, AntiSingleton, ComplexClass, LargeClass, LazyClass	11
3.	Interfacer	MessageChains, LongParameterList, SpaghettiCode, BaseClassShouldBeAbstract, ManyFieldAttributesButNotComplex, RefusedParentBequest, Blob, AntiSingleton, ComplexClass, LazyClass	10
4.	Coordinator	SpeculativeGenerality, LongParameterList, Blob, AntiSingleton, ComplexClass	5
5.	Controller	LongParameterList, SpaghettiCode, BaseClassShouldBeAbstract, LongMethod, ClassDataShouldBePrivate, Blob, AntiSingleton, ComplexClass, LazyClass	9
6.	Structurer	MessageChains, LongParameterList, BaseClassShouldBeAbstract, LongMethod, ClassDataShouldBePrivate, SwissArmyKnife, Blob, AntiSingleton, ComplexClass	8

**Table 6:** Association rules extracted from the fine-grained dataset. The results indicate the rules and their confidence values.

Rule	Confidence
LazyClass => Service Provider	0.79
ComplexClass => Interfacer	0.67
LongMethod => Service Provider	0.65
LongMethod => Interfacer	0.59
LongParameterList => Service Provider	0.54
ComplexClass, LongMethod => Service Provider	0.51
ComplexClass, LongMethod => Interfacer	0.37
ComplexClass => Service Provider	0.46
LongParameterList => Information Holder	0.37
LongParameterList => Interfacer	0.25
LongParameterList, LongMethod => Service Provider	0.11
LongParameterList, ComplexClass => Service Provider	0.09

Table 1 shows the description of the selected projects including their release, their domain, release version, and total Lines of Code (LoC) and the domain to which each project belongs (desktop or mobile). The total LoC was computed using a freely available, lightweight tool called Count Lines of Code (CLOC).^1^ CLOC counts blank lines, comment lines, and physical lines of source code.

We present a sample output of the fine-grained dataset in Table 2. The goal was to extract class names and the corresponding design smell detected in that class. The dataset consists of 23 columns (including index column) and 2513 rows which represent the total number of Java classes obtained for the selected projects. The “FullClassPath” column was extracted from the design smells detection raw files and role-stereotypes classification data respectively. The “SubClassPath” column was derived from the “FullClassPath” column and used to “inner join” design smells and role-stereotypes preprocessed data. The dataset consists of 18 design smells detected using the Pattern Trace Identification, Detection, and Enhancement in Java (Ptidej) tool^2^ [2]. These design smells include; LongMethod, ComplexClass, LongParameterList, BaseClassShouldBeAbstract, SpeculativeGenerality, ClassDataShouldBePrivate, ManyFieldAttributesButNotComplex, MessageChain, SpaghettiCode, RefusedParentBequest, SwissArmyKnife, Blob, AntiSingleton, LargeClass, LazyClass. The design smells columns contain a value of 1 or 0, which indicate the presence or absence of that design smells in a given Java class respectively.

The dataset is also classified into six role-stereotypes classification taxonomy i.e. Service Provider, Controller, Structurer, Interfacer, Coordinator and Information Holder as shown in the ``label'' column. The last column of our dataset represents the cluster in which each class belongs. The clusters were constructed using Powered Outer Probabilistic Clustering (POPC) [1] algorithms. The clustering information is useful for determining the group of design smells that often co-occur in a given role-stereotype.

## 2 Experimental Design, Materials and Methods

The process of constructing the dataset was conducted as follows.

### 2.1 Preprocessing design smells data

For the preprocessing task, we passed the project class files as input to the Ptidej tool [2] for the task of design smell detection. The tool is an open-source Java-based reverse engineering tool suite that includes several identification algorithms for idioms, micro-patterns, design patterns, and design defects [2]. Using this tool, we were able to detect eighteen (18) design smells across the selected projects. Design smells were detected and stored in “.ini” files. The file names are tagged with a specific design smell type. For example, in the K-9 Mail project, “AntiSingleton” design smell is stored as *“DetectionResults in K9 for* ***AntiSingleton****.ini"*. Fig. 1 shows sample content of “.ini” files in its raw format. Our goal was to extract class names and the corresponding design smell detected in that class.

We apply heuristics to determine the structure and pattern of class names in the detected design smells files. Regular expressions were used to extract class names and associate them with respective design smells type. The regular expressions applied to each project are listed in Table 3. A replication package for the task of design smells preprocessing is available on Zenodo as a citable GitHub repository [6].

### 2.2 Preprocessing role-stereotypes data

The processing of role-stereotype data was based on the replication package offered by Nurwidyantoro et al. [3] and can be directly accessed here.^3^ First, the selected project source code is passed to srcML,^4^ a lightweight, highly scalable, robust, multi-language parsing tool to convert source code into an XML format. Fig. 2 shows the sample output of the srcML tool. Next, we built unlabeled data consisting of 21 features for each project using code provided in the replication package^4^. In this study, the feature extraction task was carried out as follows;

1. Create a srcML representation of the source code. The output of srcML tool is a list of source code classes in a standardized XML format.
2. We use multiple XPath queries to obtain the features of interest.

The detailed steps of the features extraction are elaborated in the work of Nurwidyantoro et al. [3]. Finally, the unlabeled data was classified to one of the role-stereotype categories i.e. Service Provider, Information Holder, Interfacer, Controller, Coordinator and Structurer. The classification was achieved using the Random Forest classifier which obtained the best classification result as described by Nurwidyantoro et al. [3]. A separate repository containing source code and step by step guide for feature extraction and classification can be accessed in this GitHub repository [6].

### 2.3 Integrating design smells and role-stereotypes data

The fine-grained dataset is obtained through systematic integration of the preprocessed design smells and role-stereotype data. We created a column with unique entries called “subclasspath” in both design smell and role-stereotype data. The “subclasspath” column is derived from the full classpath and ensures that every record in that column is unique. After that,the new column was used to “inner join” design smells with role-stereotypes data. At this point,all the role-stereotypes classification features were removed to include only the classification labels. As shown in Table 2, the design smells data is also added.

#### 2.3.1 Integration using clustering

The fine-grained dataset was clustered using Powered Outlier Probabilistic Clustering (POPC) to analyze the relationship between design smells and role-stereotypes. POPC ensures flexibility in cluster construction since we do not have to specify the number of clusters upfront. This is not possible for other clustering approaches like k-means algorithm. POPC tries to mitigate these drawbacks using back-propagation techniques. It starts by building many clusters and ends with an optimal number of clusters. The algorithm is observed to work quite well on a binary dataset and converges to the expected (optimal) number of clusters on theoretical examples as elaborated by Taraba [1].

In this study, 10 clusters were created as shown in the “cluster” column of Table 2. In order to gain more insight to the significance of the clustering task, this study presents the output of the clustering task in form of a dendogram as shown in Fig. 3.

The dendrogram in Fig. 3 represents the relationship between role-stereotypes based on the common design smells that often occur in them. It is observed that the following pairs of role-stereotypes are associated i.e. (Coordinator, Structurer), (Service Provider, Information Holder) and (Controller, Interfacer). The observed associations is an indication that those pairs/groups of role-stereotypes are often affected by similar types of design smells. In Table 4, the study presents a group of design smells identified in each role-stereotype.

#### 2.3.2 Integration using association rule mining

In order to better understand the association of design smells with role-stereotypes, the study explored an alternative approach to the clustering task. The study applied the well-known Apriori algorithms [5] to construct the association rules. Association rule discovery is an unsupervised learning technique used to detect local patterns which indicates attribute value conditions that occur together in a given dataset [5] A replication package for the task of association rule mining is also provided as a citable GitHub repository [6].

Table 6 shows the result of the association rule mining. The results from the study shows, with respective degrees of confidence, the association of various design smells with role-stereotypes. For example, it can be observed that LazyClass, LongMethod and LongParameterList have strong association with the Service Provider role-stereotype.

## CRediT Author Statement

**Daniel Ogenrwot:** Conceptualization, Writing - original draft, Data curation; **Joyce Nakatumba-Nabende:** Methodology, Writing - review & editing; **Michel R.V. Chaudron:** Data curation, Conceptualization.

## Declaration of Competing Interest

The authors declare that they have no known competing financial interests or personal relationships which have, or could be perceived to have, influenced the work reported in this article.

## Footnotes

1 https://github.com/AlDanial/cloc/releases.

2 https://github.com/ptidejteam/v5.2.

3 http://oss.models-db.com/Downloads/EASE2019_ReplicationPackage/.

4 https://www.srcml.org/.
